# 3D‐printable phantoms for quantitative dynamic contrast‐enhanced MRI

**DOI:** 10.1002/mrm.30595

**Published:** 2025-06-16

**Authors:** M. Sulaiman Sarwar, Antoine Vallatos, Cher Hon Lau, Adam Waldman, Simone Dimartino, Michael J. Thrippleton

**Affiliations:** ^1^ Centre for Clinical Brain Sciences University of Edinburgh Edinburgh United Kingdom; ^2^ Institute for Bioengineering University of Edinburgh Edinburgh United Kingdom; ^3^ Glasgow Experimental MRI Centre, School of Psychology and Neuroscience University of Glasgow Glasgow United Kingdom; ^4^ Institute for Materials and Processes University of Edinburgh Edinburgh United Kingdom; ^5^ Edinburgh Imaging Edinburgh United Kingdom

**Keywords:** 3D printing, DCE, MRI, perfusion, permeability, phantoms

## Abstract

**Purpose:**

A novel 3D‐printed phantom design and methodology are proposed, addressing important requirements for technical validation, quality assurance, and multi‐site harmonization of quantitative DCE‐MRI measurements.

**Methods:**

Phantoms were produced by 3D‐printing (3DP) gels incorporating channels and pores as proxies for blood vessels and extravascular extracellular space, respectively. A flow circuit was designed to reproduce clinically relevant arterial input functions. Using nine gels with variable porosity and channel size, we evaluated the effect of 3DP parameters on DCE‐MRI parameters obtained using the extended Tofts model (ET). Physical gel and fitted model parameters were correlated by multiple linear regression.

**Results:**

All phantoms generated realistic arterial input functions, and tissue‐like signal enhancement curves were accurately modeled by the ET model. As hypothesized, blood plasma volume fraction, *v*_p_, was positively associated with the channel volume fraction, *v*_chan_, (*B* = 0.86, *p* < 0.001) and showed a weaker, negative association with gel porosity, *v*_pore_, (*B* = −0.18, *p* = 0.006). Vascular permeability‐surface area product, PS, was positively associated with both *v*_chan_ (*B* = 0.13 min^−1^, *p* < 0.001); and *v*_pore_ (*B* = 0.051 min^−1^, *p* < 0.001). The extravascular extracellular space (EES) volume fraction, *v*_e_, was positively associated with *v*_pore_ (*B* = 0.90, *p* < 0.001) but not *v*_chan_. Fitted parameters were reproducible (coefficient of variation 2.1%–3.2%).

**Conclusions:**

Tailorable 3D‐printed porous gel phantoms generating tissue‐mimicking DCE‐MRI signals have the potential to support validation, quality assurance, and multi‐site harmonization of quantitative DCE‐MRI.

## 1 INTRODUCTION

DCE‐MRI is widely used to assess perfusion, permeability, and other tissue properties.^1^ In this technique, a patient is intravenously injected with signal‐enhancing gadolinium‐based contrast agent (GBCA). Physiologically relevant parameters, useful for evaluating disease pathophysiology and treatment response, are extracted from measured changes in signal intensity using pharmacokinetic models, including the vascular permeability surface area product (*PS*), blood plasma volume fraction $v_{p}$, and the extravascular extracellular space (EES) volume fraction $v_{e}$.

Such parameters can be validated and applied as quantitative imaging biomarkers (QIB) with applications in clinical research, including investigating pathophysiology of cerebral small vessel diseases and dementias,^2^ grading and aiding in the prognosis of gliomas,^3^ and monitoring treatment response in oncology and inflammatory diseases such as rheumatoid arthritis.^4^, ^5^, ^6^, ^7^ Despite this, the clinical translation of these QIBs has been challenging due to variation in scanning equipment, acquisition methods, and analysis protocols, often leading to high variability in fitted parameters.^8^ For example, reported PS measurements to assess blood–brain barrier integrity can present order of magnitude variations between and within research groups.^9^, ^10^ Poor reproducibility and the difficulty of validation and multi‐site harmonization of DCE‐MRI QIBs have hindered their widespread adoption in research and clinical practice.

The validation of QIBs has been discussed by several academic, clinical, industrial, and regulatory groups.^11^ For example, the European Society of Radiology has published guidelines for the validation of QIBs, which are summarized in three key steps: (i) evaluating the precision of the QIBs across multiple sites, (ii) assessing accuracy of QIBs using a phantom or pathology (biopsy), and (iii) short‐ and long‐term clinical evaluation.^12^ In order to address the first step, several initiatives such as the QIB alliance and harmonizing brain imaging methods for vascular contributions to neurodegeneration have begun proposing guidelines for standardizing the acquisition and analysis used in multi‐site studies.^2^, ^13^ However, as noted by Cristinacce et al.,^14^ there is a need for suitable phantoms for monitoring the accuracy and reproducibility of QIB measures such as PS.

Several phantom designs have been proposed to date for use in DCE‐MRI, which we briefly summarize here: (1) The simplest DCE‐MRI phantoms comprise tubes of GBCA solution at different concentrations, permitting evaluation of linearity and temporal stability; however, because these phantoms are static, they can only probe limited aspects of the DCE‐MRI experiment.^15^ (2) The first realistic flow phantoms emulate a two‐compartment tissue, with hollow‐fiber membrane tubes representing blood vessels, and the surrounding space representing the EES.^16^, ^17^, ^18^ A benefit of this approach is that the hollow fibers are at a subvoxel scale (diameter <1 mm), and such phantoms can therefore generate clinically relevant signal enhancement curves. A disadvantage is that the fibers are randomly packed and bend easily, which limit uniformity, reproducibility, and predictability.^19^, ^20^ (3) Another class of flow phantoms mixes multiple feed streams in a chamber, and a signal is measured at the outlet.^21^, ^22^ This approach allows tissue‐like signal enhancement curves to be simulated, has been shown to be reproducible, and has potential for harmonization of DCE‐MRI; however, it does not probe the ability of DCE‐MRI to measure tissue properties because the phantoms do not replicate the underlying mechanisms of contrast agent transport in vivo. (4) Another type of phantom consists in generating a dynamic MRI signal as GBCA enters a chamber via a semi‐permeable membrane.^23^, ^24^ A benefit of this approach is that the concentration can be verified using liquid chromatography‐mass spectrometry, and this data can be used to correct MRI‐based concentration estimates. Limitations are that the phantom does not emulate the distribution and transport of GBCA in vivo and does not generate realistic tissue signal‐time curves.

There is, therefore, a need for reproducible subvoxel scale two‐compartment phantoms that are simple to characterize, controllable, and generate a tuneable and reproducible tissue‐like signal‐time curves. These would allow straightforward evaluation of the entire DCE‐MRI pipeline from data acquisition to estimation of pharmacokinetic parameters, and thereby support validation and harmonization of DCE‐MRI QIBs.

The aim of this work was to evaluate whether recent developments in the field of 3DP enable fabrication of controllable phantoms that mimic in vivo DCE‐MRI measurements. We present a novel phantom design consisting of a 3D‐printed (3DP) gel, incorporating sub‐mm channels and sub‐μm pores to mimic capillary blood plasma and EES compartments, respectively, at a subvoxel scale. The phantom is integrated into a flow circuit designed to generate realistic arterial input functions (AIFs). We printed nine phantoms with different material properties, hypothesizing that they would (i) replicate a clinically relevant AIF, (ii) exhibit repeatable tissue‐mimicking signal enhancement curves that can be fitted using a standard pharmacokinetic model, and (iii) yield measured pharmacokinetic parameters that are predicted by the gel porosity and the channel volume fraction.

## 2 METHODS

### 2.1 3D‐printing gel materials

Cyclohexanol (Sigma‐Aldrich, St. Louis, MO), dodecanol (Sigma‐Aldrich), polyethylene glycol diacrylate (average MW 200 g/mol, Arkema‐Sartomer, Colombes, France), alkoxylated pentaerythritol tetraacrylate (Arkema‐Sartomer), 2‐(acryloyloxy)ethyl trimethylammonium chloride solution (80% w/w, Sigma‐Aldrich), Omnirad 819 (Sigma‐Aldrich), and Tinuvin 326 (BASF, Ludwigshafen, Germany) were used to formulate 3D‐printing resins. W2P SolFlex Prototype Clear (W2P Engineering, Vienna, Austria) was used in fabricating ancillary equipment for the phantom. Isopropanol (Sigma‐Aldrich) and ethanol (Fisher Scientific, Hampton, NH) were used in the post‐processing and cleaning of 3DP parts.

### 2.2 Gel design and fabrication

**FIGURE 1:**
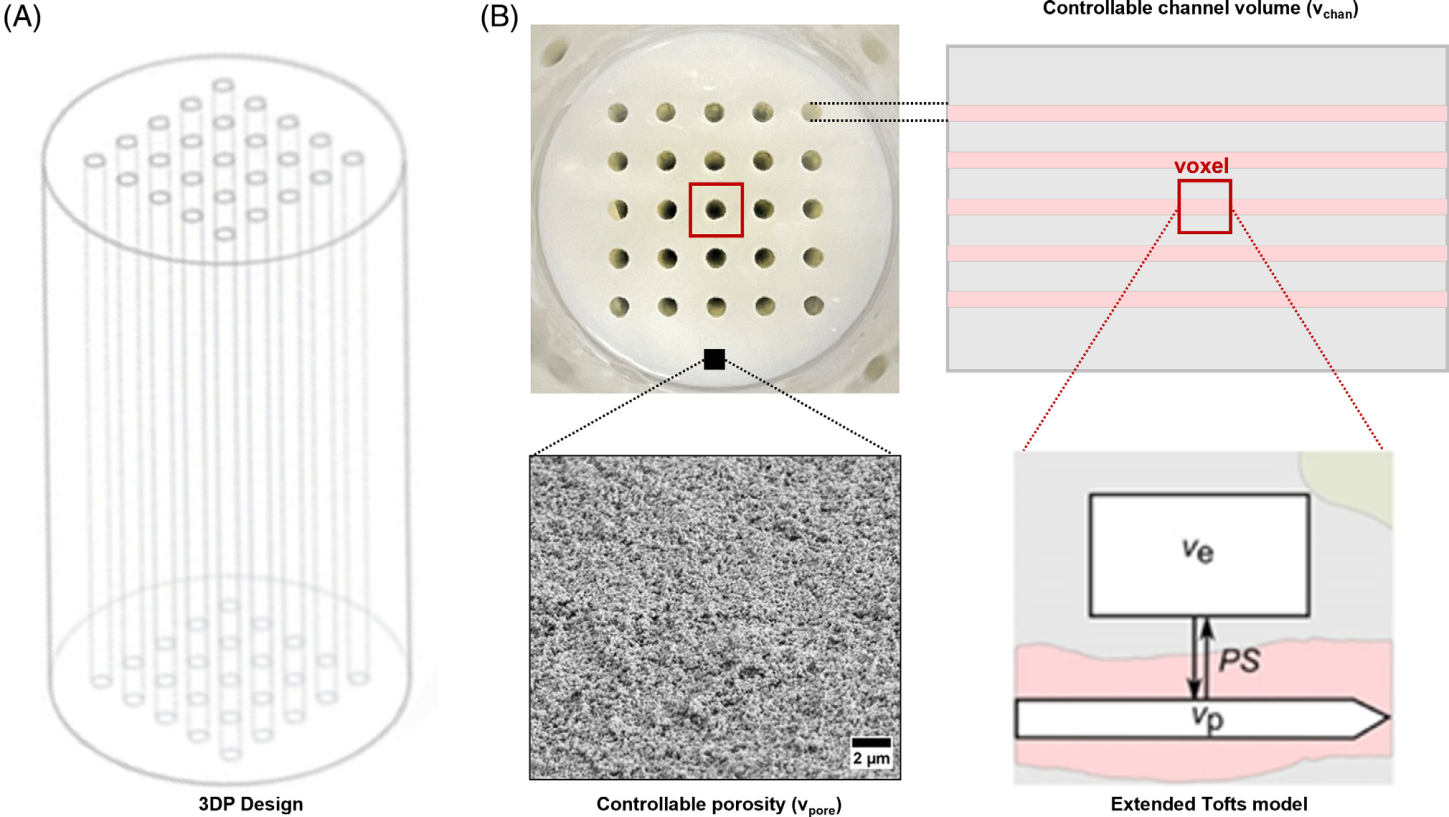
Phantom design. (A) CAD model used for 3D printing. (B) Cross‐section of 3D‐printed gel with the minimum voxel size for a DCE experiment highlighted (in red). Side view highlights the phantom's controllable channel volume (reflecting blood plasma volume) and the position of the voxel reproducing the ET model used to fit data from this two‐compartment phantom. Scanning electron microscopy image of 3D‐printed resin material with MF_pore_ = 0.60 highlights the phantom's controllable porosity (reflecting EES)^25^. CAD, computer‐assisted design; EES, extravascular extracellular space; MF_pore_, porogen mass fraction.

**FIGURE 2:**
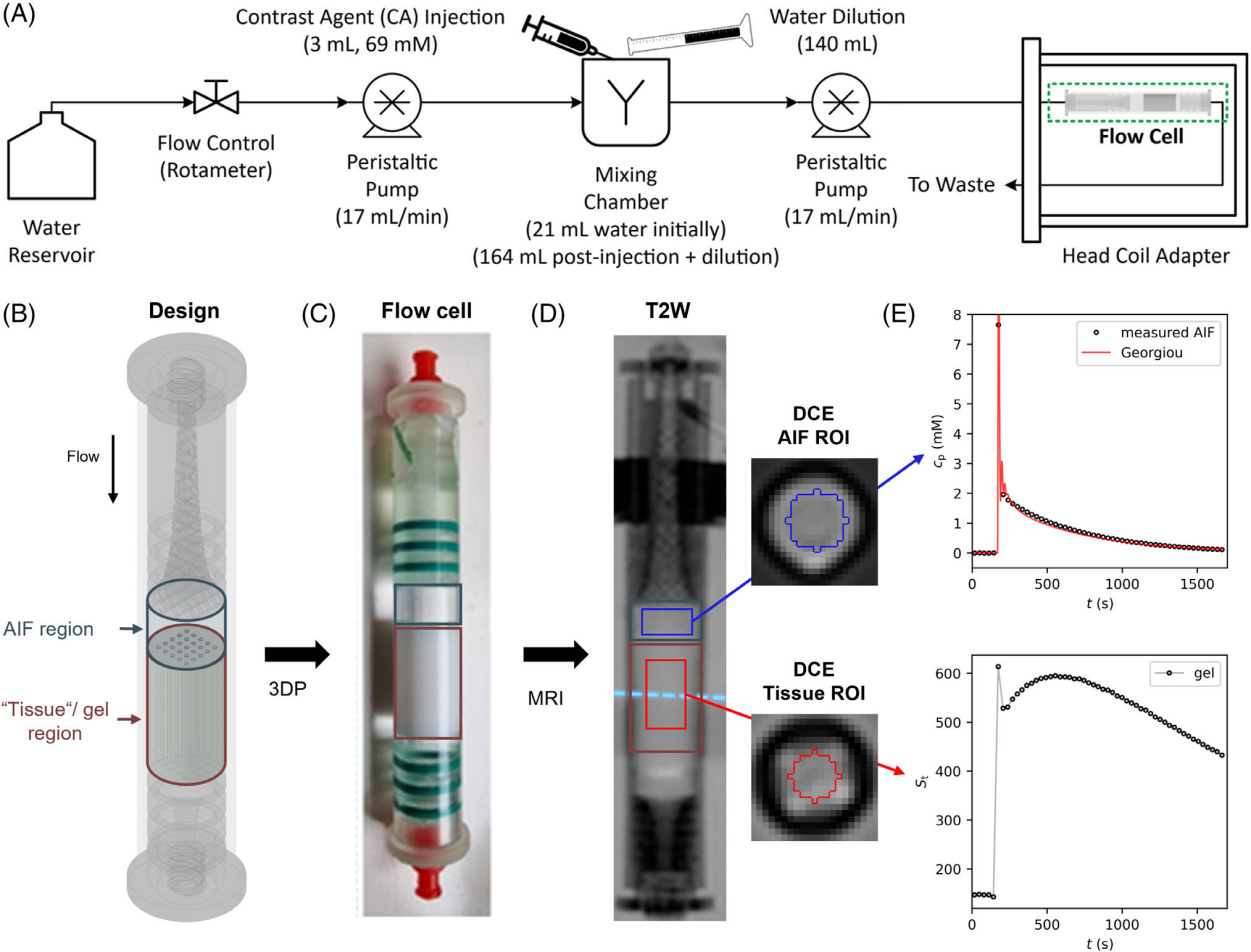
(A) Diagram of flow circuit. Water is pumped (17 mL/min) through flow circuit, with a rotameter used to regulate flow rate between two peristaltic pumps. During DCE‐MRI, contrast agent (3 mL, 69 mM) is injected into the mixing chamber (containing 21 mL water), followed 10 s later by rapid water dilution (140 mL). A picture of the flow circuit is provided in Figure S1. (B) Flow cell CAD with key regions highlighted. (C) The same regions highlighted in a photograph of the resulting 3D‐printed gel ($v_{\text{chan}}$ = 0.14, ${\mathrm{MF}}_{\text{pore}}$ = 0.70) and flow cell. (D) 3 T T_2_W image through the center of the flow cell highlighting the AIF and tissue regions, with corresponding cross sections from post‐contrast 3 T T_1_w DCE‐MRI showing AIF and tissue ROIs. (E) Measured AIF GBCA concentration (c_p_) and representative AIF function of Georgiou et al.,^27^ and measured tissue signal over the same period. AIF, arterial input functions; GBCA, gadolinium‐based contrast agent; ROI, region of interest.

The phantom comprises a tissue‐mimicking 3DP gel (Figure 1) secured in a flow cell, which is integrated within a flow circuit (Figure 2) to facilitate contrast‐agent injection. The gels were designed as a cylinder (diameter 15.36 mm, length 28.00 mm) containing a 5 × 5 grid of channels representative of blood vessels. They were fabricated using a digital light‐processing (DLP) printer (SolFlex 350, W2P Engineering) as described previously.^25^ Table S1 provides the 3D‐printing resin compositions used to manufacture the phantoms. Pores emulating EES and vessel permeability were introduced into the gel by incorporating the pore‐forming agents (porogens) cyclohexanol and dodecanol within the 3D‐printing resin; increasing porogen concentration yields increased porosity and larger pores.^26^

We define two design parameters reflecting theoretical parameters of the ET model (Figure 1): channel volume fraction ($v_{\text{chan}}$), the volume fraction of channels as specified by the computer‐assisted design (CAD) model; and porogen mass fraction (${\mathrm{MF}}_{\text{pore}}$), the mass fraction of porogens used in the 3DP resin. Nine phantoms were fabricated with all combinations of $v_{\text{chan}}$ = 0.08, 0.11, and 0.14 (corresponding to channel diameters of 0.65, 0.75, and 0.85 mm, with fixed 2 mm center‐to‐center spacing) and ${\mathrm{MF}}_{\text{pore}}$ = 0.50, 0.60, and 0.70. Values were chosen based on 3DP resolution limitations and material stability considerations.

Post‐printing, the gels were immersed in isopropanol at 40°C, which was replaced daily, for 3 days. Any 3DP residue in the channels was cleared by pushing rods of matching diameter soaked in isopropanol through them. The gels were then secured in a polycarbonate tube (I.D. 16 mm, O.D. 20 mm) by swelling action due to successive 24 h immersion periods in 80%, 50%, and 20% (volume/volume) ethanol solutions. The resulting flow cell was stored in 20% (volume/volume) ethanol solution to prevent biofilm formation and flushed with water daily for 3 days before scanning.

Bespoke endcaps for the flow cell were 3D printed using W2P SolFlex Prototype Clear resin (W2P Engineering, Vienna, Austria) to integrate the flow cell within the flow circuit and provide an even distribution of contrast agent to the channels during DCE‐MRI. The flow cell was secured within a water‐filled chamber designed to fit in a head coil.

### 2.3 Gel characterization

The porosity $v_{\text{pore}}$ of each gel was quantified by measuring wet (in water) and dried (under hot air at 70°C for 12 h) weights of seven 3DP discs (diameter 15.36 mm, length 4.00 mm):

$$v_{\text{pore}}=1-\frac{\mathrm{Dry}\;\text{Weight}}{\mathrm{Wet}\;\text{Weight}}.$$

Gels were imaged using high‐resolution T_2_‐weighted images acquired on a Bruker Pharmascan 7 T/16 cm system controlled by Paravision 5.1 software (Bruker BioSpin, Bruker, Ettlingen, Germany) with a gradient coil insert (internal diameter = 90 mm, 300 mT/m) and a four‐channel phased‐array surface receive coil. T_2_‐weighted imaging was performed using a rapid acquisition with relaxation enhancement sequence (TR/TE = 6655/52 ms, acquisition matrix size 240 × 320 × 20, 0.063 × 0.083 × 1.000 mm^3^ resolution).

### 2.4 Flow circuit

A flow circuit (Figure 2A) was used to mimic an in vivo AIF. Water was pumped (17 mL/min flow rate, Peristaltic Pump P‐230, Harvard Apparatus, Holliston, MA) from a 2 L bottle to a mixing chamber containing 21 mL of water, and from there pumped (17 mL/min flow rate, Peristaltic Pump P‐230, Harvard Apparatus) to the flow cell inside the scanner through 7 m of tubing (I.D. 1.85 mm). Water exiting the flow cell was sent to waste. At the beginning of the fourth DCE‐MRI volume acquisition, 3 mL gadobutrol (1 M Gadovist, Bayer AG, Leverkusen, Germany, diluted to 69 mM) was injected into the mixing chamber, followed 10 s later by rapid dilution with 140 mL of water. This protocol was designed and optimized to reproduce the representative AIF function described by Georgiou et al.^27^ (Figures 2D,E and S3).

### 2.5 MRI data acquisition

For all phantoms, pre‐contrast $T_{1}$ ($T_{10}$) and DCE‐MRI measurements were acquired using a 3 T Magnetom Skyra MRI scanner (Siemens Healthineers, Erlangen, Germany) using a 32‐channel phased‐array head coil. To assess repeatability, one phantom ($v_{\text{chan}}$ = 0.14, ${\mathrm{MF}}_{\text{pore}}$ = 0.70) was scanned four times, with a 2 h washout period between each scan, during which water was pumped through the phantom to eliminate residual contrast agent. The same phantom was scanned again using a second MRI scanner (3 T Siemens Magnetom Prisma, Siemens Healthineers) using a 32‐channel phased‐array head coil and the same experimental protocol.

$T_{10}$ maps were measured with voxel‐wise correction for (and estimation of) flip angle (FA) error ($k_{\mathrm{FA}}$, where $k_{\mathrm{FA}}$ is the transmitted FA divided by the nominal FA), using the driven equilibrium single pulse observation of T1 mapping with high‐speed incorporation of RF field inhomogeneities (DESPOT1‐HIFI) method^28^ (axial 3D acquisition, acquisition matrix size 96 × 96 × 96, in‐plane parallel imaging acceleration factor 2, 2‐mm isotropic resolution interpolated to 1 × 1 × 2 mm^3^), consisting of one inversion‐recovery spoiled gradient recalled echo (SPGR) acquisition (TR/TE/TI_eff_/echo spacing = 808/1.58/600/4.3 ms, FA = 5°), followed by two SPGR sequences with variable flip angle (TR/TE = 4.3/1.58 ms, FA = 2°, 12°).

DCE‐MRI was acquired with a 3D axial $T_{1}w$ SPGR sequence (TR/TE = 3.44/1.68 ms, FA = 15°, acquisition matrix size 96 × 96 × 96, 2‐mm isotropic resolution interpolated to 1 × 1 × 2 mm^3^). Fifty‐three volumes, including three pre‐contrast volumes, were acquired with a temporal resolution of 31.7 s over 28 min.

### 2.6 DCE‐MRI processing

DESPOT1‐HIFI and DCE‐MRI images were converted from Digital Imaging and Communications in Medicine (DICOM) to Neuroimaging Informatics Technology Initiative (NIFTI) format (MRIcroGL v.20; https://www.nitrc.org/projects/mricrogl/). $B_{1}^{+}$‐corrected $T_{1}$ and $k_{\mathrm{FA}}$ parametric maps were derived as described previously.^29^ Median signal intensity of the AIF region of interest (ROI) was selected using a 10 mm diameter cylindrical mask with 4 mm length located 4 mm from the gel entrance to reduce partial volume effects (Figure 2D). Median signal intensity of the gel ROI was selected using an 8 mm diameter cylindrical mask with 20 mm length centered on the gel and corresponding to 490 voxels. Signal enhancement curves were calculated relative to the mean signals of the first four volumes (before GBCA arrived in the phantom). Tissue concentration (*C*_t_) and AIF GBCA concentration (*c*_p_) curves were estimated based on the SPGR signal equation and assuming linear dependence of $1/T_{1}$ on concentration. Tissue concentration curves were fitted with the ET model under the fast‐water‐exchange limit to estimate $v_{p}$, PS, and $v_{e}$. The first four postinjection tissue concentrations were omitted from the fitting to minimize the impact of blood plasma flow rate.^30^ DCE‐MRI processing was repeated using a voxel‐by‐voxel approach. Processing was performed using SEPAL v1.3.2 (https://github.com/mjt320/SEPAL), which has been benchmarked through the International Society for Magnetic Resonance in Medicine Open‐Science Initiative for Perfusion Imaging.^31^ Full details and Jupyter Python 3.11.4 notebooks for the processing can be found in the open‐access repository (https://doi.org/10.7488/ds/7944).

### 2.7 Statistical analysis

Results are given as mean ± 1 SD unless noted otherwise. To investigate the effect of the phantom parameters ($v_{\text{chan}}$, $v_{\text{pore}}$) on the fitted pharmacokinetic parameters ($v_{p}$, PS, and $v_{e}$), the former were used as predictors for the latter in multiple linear regression models with intercepts. The coefficients (*B*) and *p*‐values (*p*) are reported; the predictive power of the models was reported as the root mean square error (RMSE) of the predicted versus measured parameter values. Full details of the statistical analyses are provided in Jupyter notebooks in the open‐access repository.

### 2.8 Accessing data

DESPOT1‐HIFI, DCE‐MRI, and high‐resolution 7 T T_2_‐weighted images are available in an open access‐repository (https://doi.org/10.7488/ds/7944), which includes the scripts used to process the images.^31^

## 3 RESULTS

### 3.1 Gel physical parameters

**FIGURE 3:**
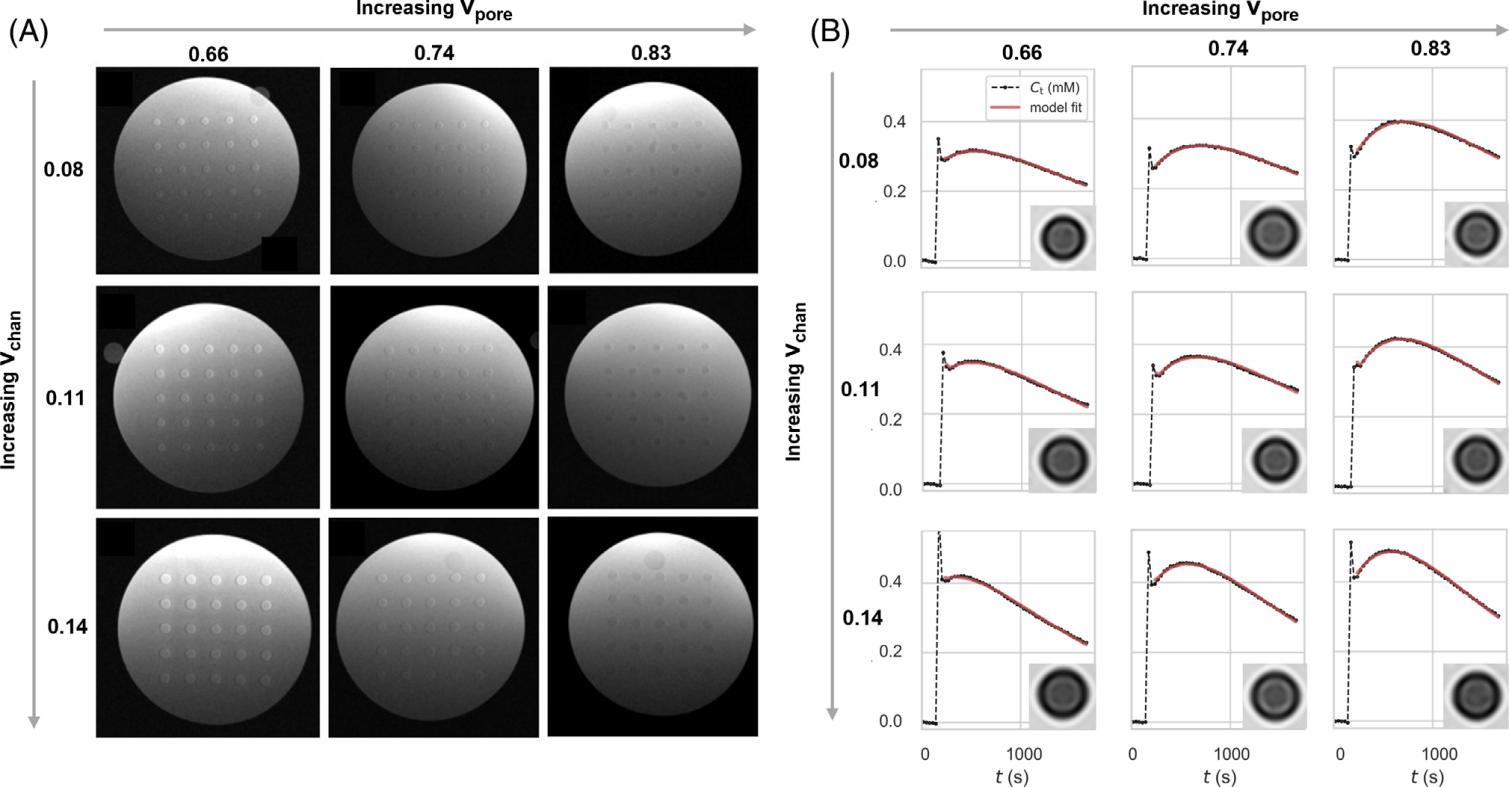
(A) 7 T T_2_W images of the gel cross section for varying gel parameters ($v_{\text{chan}}$ and $v_{\text{pore}}$), and (B) resulting Gd concentration curves for each gel fitted with the ET model (with inlay of the corresponding phantom cross‐section in the post‐contrast 3 T T_1_w DCE‐MRI). Gd, gadolinium; *v*_pore_, material porosity.

Gel porosity, $v_{\text{pore}}$, was measured using the dry/wet weight method on seven printed discs for each porogen mass fraction (${\mathrm{MF}}_{\text{pore}}$). Gels with ${\mathrm{MF}}_{\text{pore}}$ equal to 0.5, 0.6, and 0.7 had $v_{\text{pore}}$ of 0.664 ± 0.016, 0.747 ± 0.001 and 0.825 ± 0.002, respectively. These results confirm the expected linear relation between ${\mathrm{MF}}_{\text{pore}}$ and $v_{\text{pore}}$ (Figure S2). The variation in channel size and center‐to‐center spacing for each gel was evaluated using high‐resolution 7 T $T_{2}w$ images (Figure 3A), confirming that inter‐channel spacing and *v*_chan_ were within the expected ranges (Figure S3).

### 3.2 Phantom AIFs

The flow circuit was optimized to reproduce a representative clinical AIF model.^27^ AIFs measured in all experiments were compared with the target population‐averaged AIF (Figure S4). Aligned AIF curves were reproducible with a maximum SD of 0.64 mM at peak concentration and 0.01–0.07 mM subsequently.

### 3.3 Phantom DCE‐MRI signals and pharmacokinetic modeling

We investigated whether the nine phantoms with different *v*_pore_ and *v*_chan_ would yield tissue‐like DCE‐MRI time courses and tissue concentration curves (Figure 3B) that could be fitted using the ET model. With increasing $v_{\text{chan}}$, *C*_t_ is seen to increase, particularly during the early part of the time course. With increasing $v_{\text{pore}}$, the peak *C*_t_ (after the first pass) is increased, and the characteristic “wash‐in–washout” curve shape becomes more pronounced.

**FIGURE 4:**
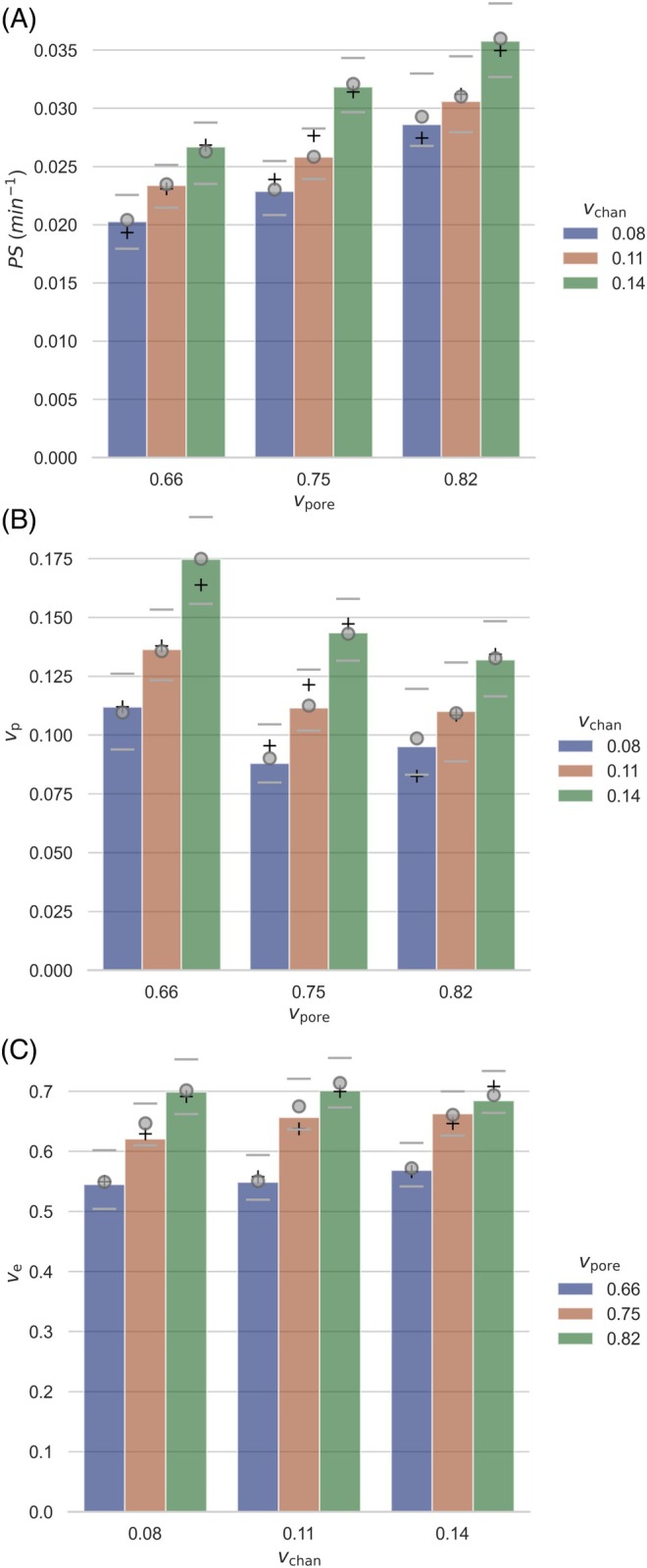
Extended Tofts model fitting results for each of the nine phantoms, arranged according to the phantom design parameters ($v_{\text{chan}}$ and $v_{\text{pore}}$). The bars show (A) PS, (B) $v_{p}$, and (C) $v_{e}$ results for the ROI analysis. Median and 25th/75th percentile voxelwise results are shown as circles and dashes, respectively. Values predicted by the multiple linear regression models are shown as “+”. PS, permeability surface area; ROI, region of interest.

The ET model closely fitted the *C*_t_ curves for all nine phantoms (Figure 3B), yielding values of *PS*, $v_{p}$, and $v_{e}$ for each phantom (Figure 4). Multiple linear regression models strongly predicted *v*_p_ (RMSE 0.0071), *PS* (RMSE 0.00094 min^−1^), and *v*_e_ (RMSE 0.013). *v*_p_ was positively associated with *v*_chan_ (*B* = 0.86, *p* < 0.001) and showed a weaker, negative association with *v*_pore_ (*B* = −0.18, *p* = 0.006). *PS* was positively associated with both *v*_chan_ (*B* = 0.13 min^−1^, *p* < 0.001) and *v*_pore_ (*B* = 0.051 min^−1^, *p* < 0.001). *v*_e_ was positively associated with *v*_pore_ (*B* = 0.90, *p* < 0.001) but not *v*_chan_. Voxelwise fitting of the DCE‐MRI data yielded very similar pharmacokinetic parameter values (Figure 4).

### 3.4 Repeatability and reusability

To assess repeatability and reusability of the phantoms, one gel ($v_{\text{chan}}=0.14$, $v_{\text{pore}}=0.82$) was scanned four times over 2 days, with a minimum 2 h washout period between scans. The resulting pharmacokinetic parameters are shown in Figure S5 and had coefficients of variation of 3.2% (*v*_p_), 2.2% (*PS*), and 2.1% (*v*_e_). The 2 h washout period was shown to be sufficient for the mean signal intensity in the gel ROI to return to baseline (Figure S6). Scanning the same phantom with a second 3 T scanner yielded pharmacokinetic parameters consistent with those obtained using the primary scanner (Figure S5).

## 4 DISCUSSION

The aim of this work was to evaluate whether recent developments in 3D printing enable fabrication of controllable phantoms that mimic tissue DCE‐MRI signals for validation and harmonization of DCE‐MRI. We found that the tissue‐mimicking gels do generate tissue‐like signal enhancement curves that are determined by the specified channel size and gel porosity, and can be fitted using a standard pharmacokinetic model.

### 4.1 Physical characterization of 3DP phantoms

An advantage of the 3D‐printing approach presented here is that physical properties of the phantom such as channel size and gel porosity can be easily modulated through the CAD model and resin chemistry, respectively. As expected, the porogen mass fraction (${\mathrm{MF}}_{\text{pore}}$) is linearly associated with the measured physical porosity ($v_{\text{pore}}$) of the printed gel; thus, a key aspect of the phantom microstructure was shown to be controllable. The difference in absolute values of MF_pore_ and *v*_pore_ is expected due to polymerization‐induced phase separation effects during the curing of the parent liquid resin.^32^ The coefficient of variation in porosity was below 2.3%, reflecting the high precision and low batch‐to‐batch variability associated with 3DP methods. High‐resolution 7 T $T_{2}w$ imaging was used to evaluate the 3DP quality, confirming the approximate channel size and center‐to‐center spacing for each phantom to be approximately as specified by the CAD model. Despite the fact the MRI lacked the resolution (compared to 3DP) to provide with a high‐precision assessment, these experiments allowed to validate the inter‐channel gap and observe the expected linear relation between the nominal channel diameter and the measured diameter, $v_{\text{chan}}$ (Figure S2).

### 4.2 Representative clinical AIF

We successfully developed a flow circuit to generate a clinically relevant AIF, choosing that described by Georgiou et al.^27^ The flow circuit system was robust and reproducibly generated the AIF function in all nine phantoms, confirming our first hypothesis that the phantoms would replicate a clinically relevant AIF.

Future improvements to the flow circuit could be made to further reduce variability. The dispersion of GBCA could be reduced by coiling the tubing,^33^ and there is scope to automate injection of contrast agent and water dilution. In addition, the current system is designed to generate AIF models at relatively low temporal resolution (>10 s), where first‐pass and secondary GBCA concentration peaks are not fully resolved. If necessary, this limitation could be addressed through the addition of recirculation loops within the circuit. We note that AIFs vary between individual patients, organs, and regions, and are subject to delay and dispersion effects; the flow circuit used here is based on simple fluid dynamic principles and the AIF can be varied by adjusting the timing and flow circuit parameters (i.e., pump flow rate, the initial and diluted mixing chamber volumes, injected gadolinium concentration and volume).

### 4.3 Tissue‐mimicking signal enhancement curves

The phantoms described in this report are unique due to having two controllable subvoxel compartments: a channel space, which is modulated through the design of the CAD model; and a porous space, which is modulated through the formulation of the 3D printing resin. As a result, all nine phantoms generated tissue‐like “wash‐in–washout” signal‐time profiles, including vascular and EES contributions. All nine datasets could be fitted closely using the widely used two‐compartment ET pharmacokinetic model. Further experiments confirmed the reusability and repeatability of the phantom. These findings confirm our second hypothesis that 3DP phantoms can exhibit repeatable tissue‐mimicking signal uptake curves matching a standard pharmacokinetic model.

### 4.4 Controllable DCE‐MRI parameters

We demonstrated that the phantoms' physical parameters $v_{\text{chan}}$ and $v_{\text{pore}}$, which are modified via the manufacturing process, are strong predictors of the measured DCE‐MRI model parameters. *v*_p_ was primarily determined by *v*_chan_, consistent with our intention to mimic blood vessels using printed channels. The weaker, negative association with *v*_pore_ is expected because increasing the pore volume increases the overall volume of water in the gel, thereby reducing the fractional volume of water within the channels. Similar behavior has been reported in hollow fiber phantoms.^34^, ^35^

*PS* was positively associated with both phantom parameters. This finding is expected because *v*_chan_ directly affects *S* (surface area of vessels per unit tissue volume), whereas increasing *v*_pore_ results in larger or more gaps in the channel walls. Increases in either parameter are therefore expected to allow faster leakage of GBCA from the channels to the surrounding porous gel.

Finally, *v*_e_ was determined by *v*_pore_, consistent with our design in which pores of the gel emulate the EES. These findings confirm our third hypothesis that the controlled physical parameters of the phantoms predict the pharmacokinetic parameters measured by DCE‐MRI. It is encouraging that *v*_chan_ and *v*_p_ (and *v*_pore_ and *v*_e_) have similar (though not equal) magnitudes and are related with coefficients close to unity.

### 4.5 Strengths and Limitations

The strengths of this work include the development of phantoms with realistic, predictable, and repeatable AIF and tissue signal curves. By adjusting the channel volume fraction (0.08–0.14) and gel porosity (0.66–0.83), we constructed nine phantoms covering a moderate range of $v_{p}$ (0.09–0.17), PS (0.020–0.036 min^−1^), and $v_{e}$ (0.54–0.70) values. Moreover, repeated experiments involving two scanners showed repeatability and, therefore, potential to act as a reference for quality assurance. In comparison to existing phantom manufacturing techniques, 3DP offers more stable, uniform, and reproducible manufacturing at high spatial resolution, which could enable the production of multiple copies of phantoms with specified properties to support technique optimization, multi‐modal comparison, and harmonization in multisite studies.

The spatial precision of 3DP facilitates the construction of phantoms designed to mimic tissue and contrast agent transport properties. For this reason, the approach also has potential applications to other perfusion and permeability imaging techniques, such as diffusion‐weighted arterial spin labeling. However, the minimum achievable channel size used in this work was limited by overcuring, that is, unwanted polymerization resulting from light scattering during 3DP, which reduces the effective printing resolution; and required manual clearing of overcured resin from the channels, which potentially risks damage to the gel. Overcuring is a complex phenomenon that depends on the 3DP hardware, the geometry of the CAD model, and the resin chemistry.^36^, ^37^, ^38^ It is expected that using a higher resolution printer will reduce overcuring. In this work, we employed a DLP printer with a resolution of 50 μm. DLP printer technology is advancing rapidly, and benchtop printers with 10 μm resolution are now commercially available.^39^, ^40^ In addition, new technologies such as grayscale printing are being developed, which can further reduce overcuring.^41^ Adoption of ultrahigh resolution DLP printers should enable the fabrication of gels containing smaller channels, and therefore facilitate phantoms with a wider range of $v_{p}$ values and support DCE‐MRI protocols with higher spatial resolution. For example, a channel size of 0.30 mm with 2 mm spacing would correspond to a channel volume fraction of 0.02, which is close to blood plasma volume fraction values measured in brain tissues.^42^

The nine manufactured gels all yielded DCE‐MRI data that could be fitted using the ET model. Though this model is commonly widely used in oncology and other fields, other models are more appropriate in other situations. For example, subtle leakage of the blood–brain barrier is typically probed using the Patlak model because permeability is low and backflux is negligible. Whereas our phantom design permits manipulation of the pharmacokinetic parameters, these are constrained by the nature and range of the manufacturing parameters. For example, PS can be reduced by decreasing the gel porosity, but this has the additional effects of increasing $v_{p}$, reducing $v_{e}$ and reducing the overall water content. We are exploring other approaches to increase the degrees of freedom and design flexibility. For example, incorporating a membrane with controllable permeability via interfacial polymerization^43^ could facilitate adjustment of PS independently of $v_{p}$ and $v_{e}$, further expanding the range of tissue properties and pharmacokinetic models that can be probed.

A further limitation applying to all DCE‐MRI phantoms is that ground truth is difficult to define and determine. A strength of our phantom design is that it has well‐controlled ground‐truth *physical* parameters (e.g., channel diameter, porosity), which can be specified at production and verified through drying experiments, microscopy, and high‐resolution imaging. However, pharmacokinetic parameters are defined within a simplified mathematical model, are usually measured by DCE‐MRI, and are subject to its limitations (e.g., temporal resolution, assumptions of fast water exchange, and instantaneous mixing). For example, ground‐truth *PS* cannot easily be derived from the physical structures of our phantoms, although computer simulations or alternative imaging methods could be used to probe the relationships. We nevertheless argue that the strong and physically plausible associations between physical and pharmacokinetic parameters demonstrated in this work support the claim of DCE‐MRI to probe specific tissue properties. For quality assurance purposes in clinical research, a pragmatic approach is suggested, for example, by monitoring phantom imaging measurements over time and across sites.

## 5 CONCLUSIONS

We developed a novel approach to constructing DCE‐MRI phantoms, incorporating a clinically relevant AIF and highly controllable tissue‐mimicking materials. Measured DCE‐MRI parameters were repeatable and predicted by the physical properties of the phantoms. 3DP gel phantoms have the potential to address some of the limitations of previous phantom designs and to support the application of quantitative DCE‐MRI biomarkers in research and clinical practice.

## FUNDING INFORMATION

M.S.S., A.V., and this work were mainly funded by the Engineering and Physical Sciences Research Council (EPSRC), grant EP/T517884/1. M.J.T. was funded by the Scottish Chief Scientist Office (CSO) through the National Health Service (NHS) Lothian Research and Development Office. Financial support was also provided by the Brain Tumour Charity and the Clerk Maxwell Cancer Research Fund.

## Supporting information


**Figure S1.** Flow circuit experimental set up.
**Figure S2.** Relationship between porogen mass fraction (MFpore) and material porosity (*v*_pore_).
**Figure S3.** 3DP channel size measures.
**Figure S4.** Arterial input functions (AIF) repeatability.
**Figure S5.** Repeatability and reproducibility assessment.
**Figure S6.** CLEANING TIME ASSESSMENT.
**Table S1.** 3DP material mass fraction effect on porosity.

## Data Availability

DESPOT1‐HIFI, DCE‐MRI, and high‐resolution 7 T T_2_‐weighted images are available in an open access‐repository (https://doi.org/10.7488/ds/7944), which includes the scripts used to process the images.^31^
