# Supplementary material for: 3D‐printable phantoms for quantitative dynamic contrast‐enhanced MRI
Source: Magn Reson Med. 2025 Jun 16;94(5):1889–99. doi: 10.1002/mrm.30595 (PMC12393202; doi:10.1002/mrm.30595)

Supplemental Material for “3D-printable phantoms for quantitative dynamic contrast-enhanced MRI”

Supporting Table. S1. 3DP material mass fraction effect on porosity

Resin compositions used in this work, expressed as the mass of each component, m_i, as a fraction of the total mass of all components, m_{Total}. For all resin compositions, 0.01 g of Omnirad 819 and 0.00125 g of Tinuvin 326 were added to 100 g of monomer-porogen micture.

The gel porosity (υ_{pore}) was manipulated by altering the porogen (cyclohexanol and dodecanol) content, expressed as the porogen mass fraction, $MF_{pore} = (m_{cyclohexanol} + m_{dodecanol}) / m_{Total}$.

	$MF_{pore} = 0.50$	$MF_{pore} = 0.60$	$MF_{pore} = 0.70$
Cyclohexanol	0.40	0.48	0.56
Dodecanol	0.10	0.12	0.14
PEGDA	0.15	0.12	0.09
APETA	0.15	0.12	0.09
AETAC	0.20	0.16	0.12

Supporting Figure S1. Flow circuit experimental set up

(A) Diagram of flow circuit. Water is pumped (17 mL/min) through flow circuit, with a rotameter used to regulate flow rate between two peristaltic pumps. During DCE-MRI, contrast agent (3 mL, 69 mM) is injected into the mixing chamber (containing 21 mL water) and followed 10 s later by rapid water dilution (140 mL). (B) Photograph of flow circuit.

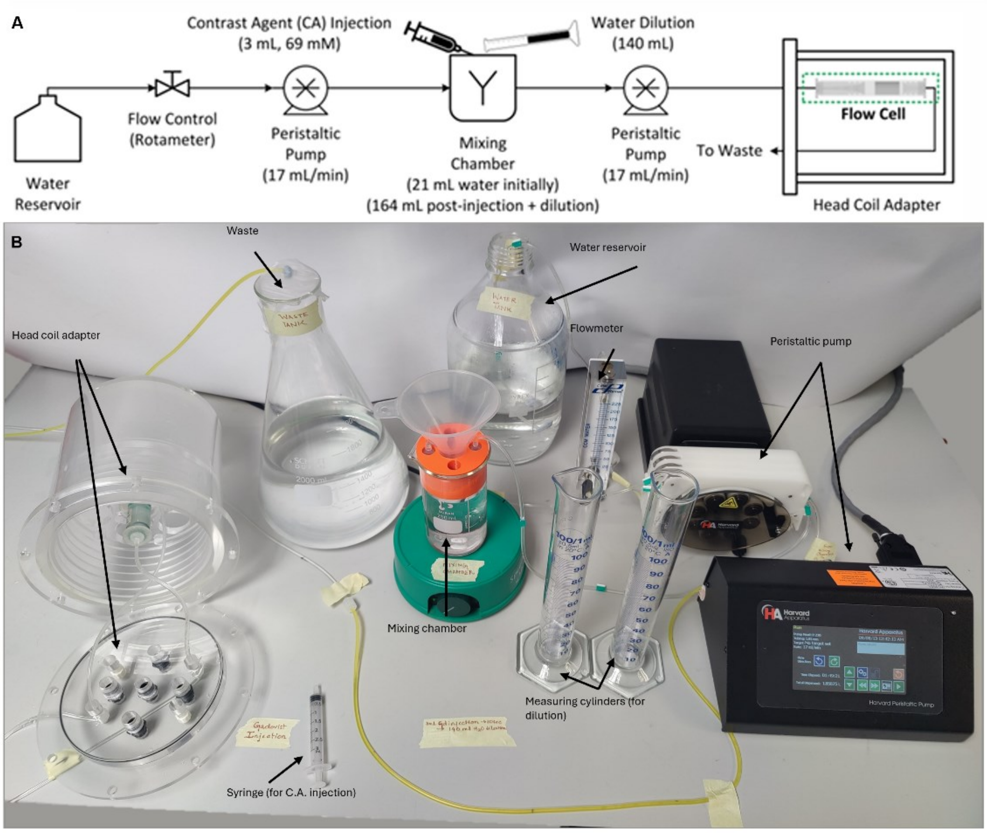

Supporting Figure S2. Relationship between porogen mass fraction (*MF_{pore}*) and material porosity (v_{pore}).

Circles and error bars indicate the mean ± 1 standard deviation of 7 v_{pore} measurements.

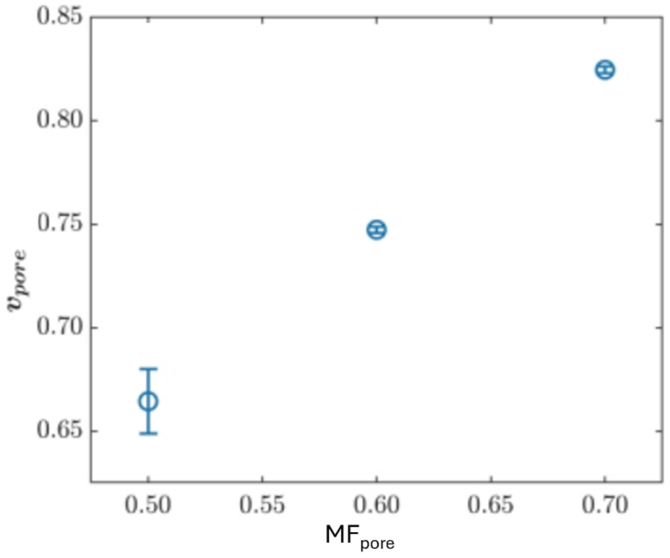

Supporting Figure S3. 3DP channel size measures

A: High-resolution 7-T MR images of the 9 phantoms with three examples of estimated channel volume fraction v_{chan} $est.$: v_{chan} ROI (red circle) on a 4x4mm inlay (MF_{pore}=0.5) and resulting values in white.

B: Measured (v_{chan} est.) vs. designed (v_{chan} CAD) channel size. Dashed line corresponds to y = x. Error bars correspond to ± 1 SD (n = 25 measures).

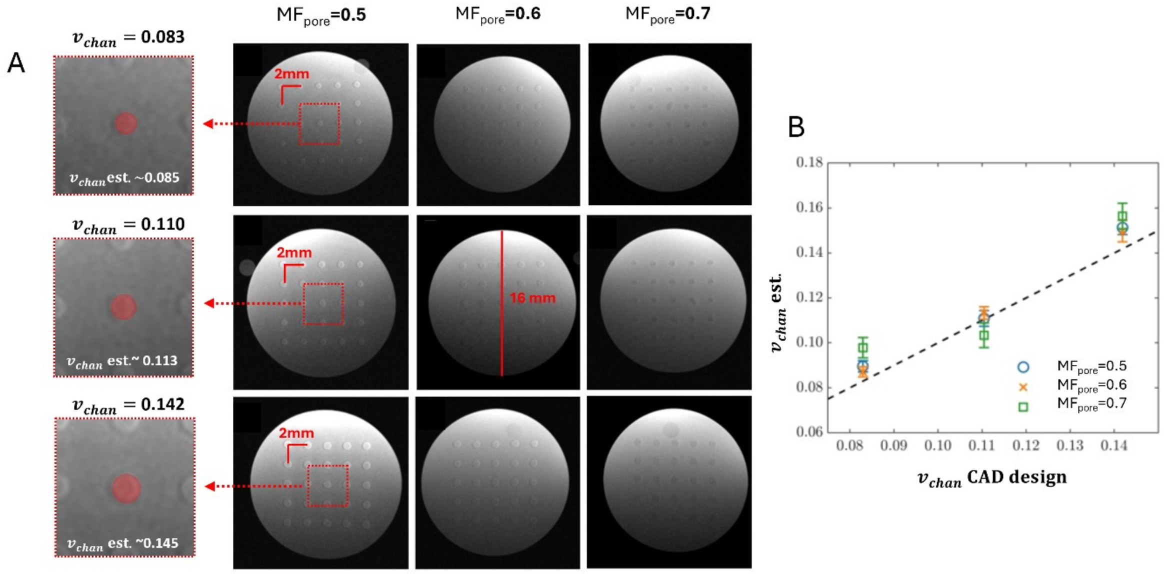

Supporting Figure S4 AIF repeatability

Measured AIF for the 12 phantom experiments conducted on the primary MRI scanner, plotted with the population AIF by Georgiou, et al.

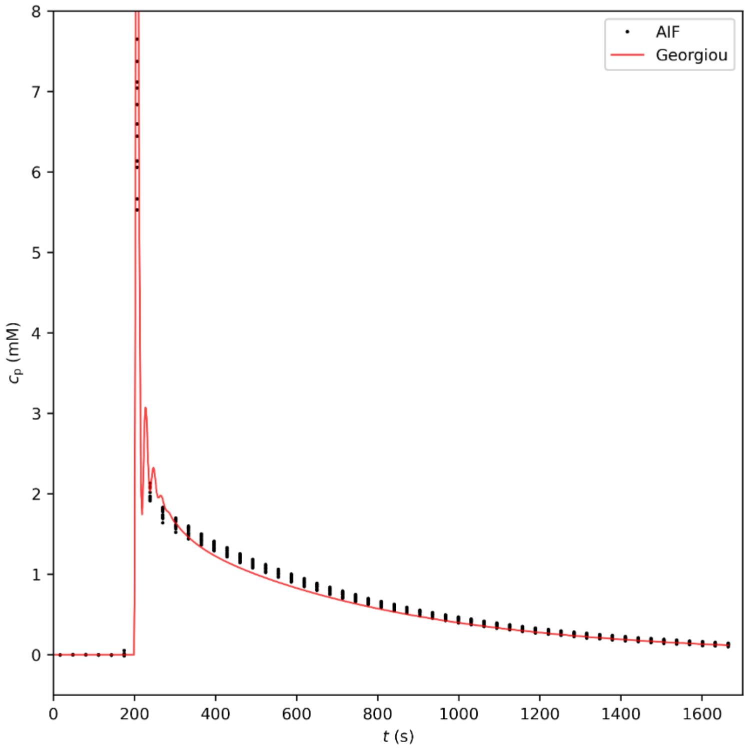

Supporting Fig. S5. ***Repeatability and reproducibility assessment***

To assess repeatability, one of the phantoms (MF_{pore} = 0.70 and v_{chan} = 0.14) was scanned a further three times following a two-hour minimum wash-out time and a complete reassembly of the experimental set up. The repeat values were compared to the overall results (c.f. main paper fig. 4), where columns/data points show DCE parameter values obtained for each experiment, with the last column (MF_{pore} = 0.70 and v_{chan} = 0.14) indicating the mean over 4 repeated experiments performed using the primary MRI scanner; results of an experiment reproduced using a second MRI scanner are shown as red data points.

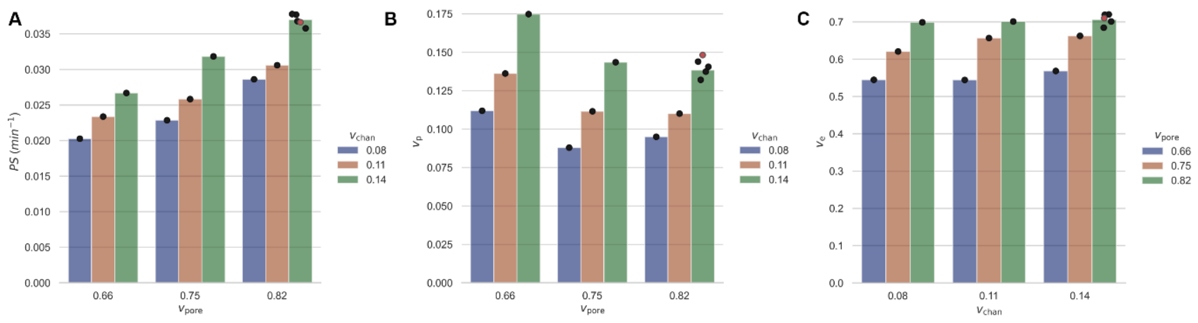

Supporting Fig. S6 Cleaning time assessment

A two-hour water wash-out period was sufficient for the signal intensity to return to baseline. Data from phantom produced under MF_{pore} = 0.70 and v_{chan} = 0.14.

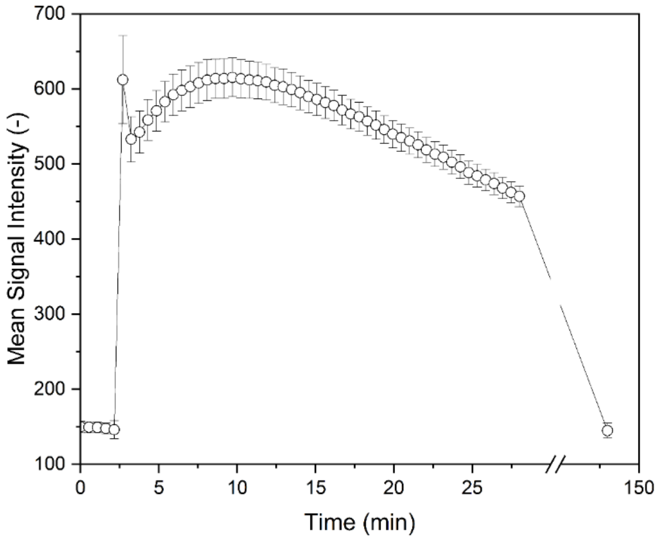

Supplement: Supplementary file 1 — Figure S1. Flow circuit experimental set up. Figure S2. Relationship between porogen mass fraction (MFpore) and material porosity (v pore). Figure S3. 3DP channel size measures. Figure S4. Arterial input functions (AIF) repeatability. Figure S5. Repeatability and reproducibility assessment. Figure S6. CLEANING TIME ASSESSMENT. Table S1. 3DP material mass fraction effect on porosity. [file MRM-94-1889-s001.pdf]
